# Characteristics and demography of low energy fall injuries in patients > 60 years of age: a population-based analysis over a decade with focus on undertriage

**DOI:** 10.1007/s00068-024-02465-3

**Published:** 2024-02-07

**Authors:** Martine A. Aarsland, Clemens Weber, Cathrine H. Enoksen, Ingvild Dalen, Kjell Egil Tjosevik, Pieter Oord, Kenneth Thorsen

**Affiliations:** 1 Section for Traumatology; Surgical Clinic, Stavanger University Hospital, Stavanger, Norway; 2 Department of Orthopaedic Surgery, Stavanger University Hospital, PO Box 8100, N-4068 Stavanger, Norway; 3 Department of Neurosurgery, Stavanger University Hospital, Stavanger, Norway; 4 Department of Quality and Health Technology, University of Stavanger, Stavanger, Norway; 5 Department of Research, Stavanger University Hospital, Stavanger, Norway; 6 Department of Emergency Medicine, Stavanger University Hospital, Stavanger, Norway; 7 Department of Gastrointestinal Surgery, Stavanger University Hospital, Stavanger, Norway; 8 Department of Clinical Medicine, University of Bergen, Bergen, Norway

**Keywords:** Trauma, Mortality, Injury severity, Traumatic brain injury, Undertriage

## Abstract

**Background:**

An increasing group of elderly patients is admitted after low energy falls. Several studies have shown that this patient group tends to be severely injured and is often undertriaged.

**Methods:**

Patients > 60 years with low energy fall (< 1 m) as mechanism of injury were identified from the Stavanger University Hospital trauma registry. The study period was between 01.01.11 and 31.12.20. Patient and injury variables as well as clinical outcome were described. Undertriage was defined as patients with a major trauma, i.e., Injury Severity Score (ISS) > 15, without trauma team activation. Statistical analysis was performed using the Chi-squared test for categorical variables and the Mann–Whitney U test for continuous variables.

**Results:**

Over the 10-year study period, 388 patients > 60 years with low energy fall as mechanism of injury were identified. Median age was 78 years (IQR 68–86), and 53% were males. The location of major injury was head injury in 41% of the patients, lower extremities in 19%, and thoracic injuries in 10%. Thirty-day mortality was 13%. Fifty percent were discharged to home, 31% to nursing home, 9% in hospital mortality, and the remaining 10% were transferred to other hospitals or rehabilitation facilities. Ninety patients had major trauma, and the undertriage was 48% (95% confidence interval, 38 to 58%).

**Conclusions:**

Patients aged > 60 years with low energy falls are dominated by head injuries, and the 30-day mortality is 13%. Patients with major trauma are undertriaged in half the cases mandating increased awareness of this patient group.

## Introduction

The organization of trauma systems is a major secondary preventive effort to reduce the impact of traumatic injuries [1]. Trauma teams are an important aspect of this system, and certain criteria exist to activate such teams [2]. The sensitivity and specificity of such criteria can lead to under and/or overtriage [1]. An undertriage rate below 5% is considered acceptable by the American College of Surgeons Committee on Trauma, while an overtriage rate of 25–50% has been deemed acceptable [2]. A high overtriage rate could strain the system due to the allocation of resources to the trauma population and may lead to worse treatment for other patient groups. A low undertriage rate could potentially lead to an improved treatment and better outcome for trauma patients. Contrary, a high undertriage rate, where the trauma system does not identify highly injured patients, may lead to a higher mortality among trauma patients [3].

Fall injuries among elderly patients are common and a major cause of mortality and morbidity [4–7]. Falls and higher age have further been associated with undertriage, which potentially can have a significant impact on treatment and outcome for the patient [8]. Low energy falls have particularly been found to have detrimental outcome for older patients with increasing rates of head injuries [9].

A recent study from our trauma center identified 50% of the undertriaged patients to be > 65 years of age, and that patients with low energy fall as mechanism of injury were more likely to be undertriaged [10]. Hence, we wanted to investigate the elderly trauma patient with low energy falls further, to possibly improve triage, treatment, and outcome for this fragile group of patients.

The aims of this study were to describe the characteristics and demography of patients aged > 60 years with low energy falls and to investigate the rate of undertriage and clinical outcome of these patients.

## Methods

### Study population

The study population were all trauma patients with low energy fall and age > 60 years included in the Stavanger University Hospital (SUH) trauma registry from 01.01.2011 until 31.12.2020. Age > 60 years is a relative trauma criteria in present guidelines; therefore, this age cutoff was chosen for this study. SUH serves as the primary trauma center for a population of about 370,000 citizens and as a trauma referral center for approximately 550,000 people living in southwestern Norway [11]. The SUH trauma registry is based on all trauma team activations (TTA) since 2003. It is supplemented by registrars who manually sift all admissions without a trauma team and add all patients with an Injury Severity Score (ISS) > 9 [12]. The registrars have been trained by an Association for the Advancement of Automotive Medicine-certified Abbreviated Injury Scale (AIS) course [13]. Patients admitted with isolated fractures and ISS ≤ 9 are handled by orthopedic surgeons only and are not included in the trauma registry. Only patients actually admitted to the hospital are included; thus, prehospital mortality is not included in this study.

### Study design

This observational cohort study is based on a prospectively maintained institutional trauma registry database covering all trauma patients admitted to SUH between January 1, 2011, and December 31, 2020. The STROBE guidelines were adhered to when appropriate [14].

### Definitions

ISS was calculated from the injuries found during admission to hospital and was registered after discharge. Major trauma was defined as an ISS > 15 [15]. Undertriage was noted when a patient with an ISS > 15 was admitted to the hospital without TTA [16]. Overtriage is defined as ISS ≤ 15 with TTA (see Box 1).

**Box 1** Definition of under and overtriage and the groups in this study.

**Table:** 

	No TTA	TTA
ISS ≤ 15	Group 1	Group 2 (overtriaged)
ISS > 15	Group 3 (undertriaged)	Group 4

*TTA*, Trauma team activation; *ISS*, Injury Severity Score

A TTA in this study includes activation of both full and reduced trauma teams.

There have been some changes of the composition of the trauma team at SUH during the study period. In the years before 2018, a two-tiered protocol with both full and reduced trauma teams was available, while the period from 01.01.2018 involves a one-tiered protocol with only full trauma teams [12]. The composition of the teams is shown in Box 2.

**Box 2** Trauma team composition.

**Table:** 

Full team	Reduced team
Team leader surgeon	Team leader surgeon
General surgeon	General surgeon
Orthopaedic surgeon	Orthopaedic surgeon
Anaesthetist	2 ED nurses
Nurse anesthetist	
Radiologist	
2 Radiographers	
3 ED nurses	
Theatre nurse	
Laboratory technician	

*ED*, Emergency department

The New Injury Severity Score (NISS) is a revised version of ISS. Both ISS and NISS are defined by the Abbreviated Injury Scale (AIS) and calculated by the three most severe injuries. ISS is calculated by the three most injured body regions, while NISS is calculated by the three most severe injuries regardless of body region [17].

### Statistical analysis

Statistical analysis was performed with STATA/SE 17.0. Descriptive statistics are presented as counts and percentages for categorical data and as medians and interquartile ranges (IQR) for continuous data. Comparisons between groups of patients were performed by the Chi-squared test (dichotomous variables) and by the Mann–Whitney U test (continuous or ordinal variables). Central findings were presented with 95% confidence intervals (CI), estimated using the OpenEpi online calculator [18]. Two-sided *p*-values < 0.05 were considered statistically significant.

### Ethics

The study has been approved by the local Data Protection Office as required.

## Results

**Fig. 1:**
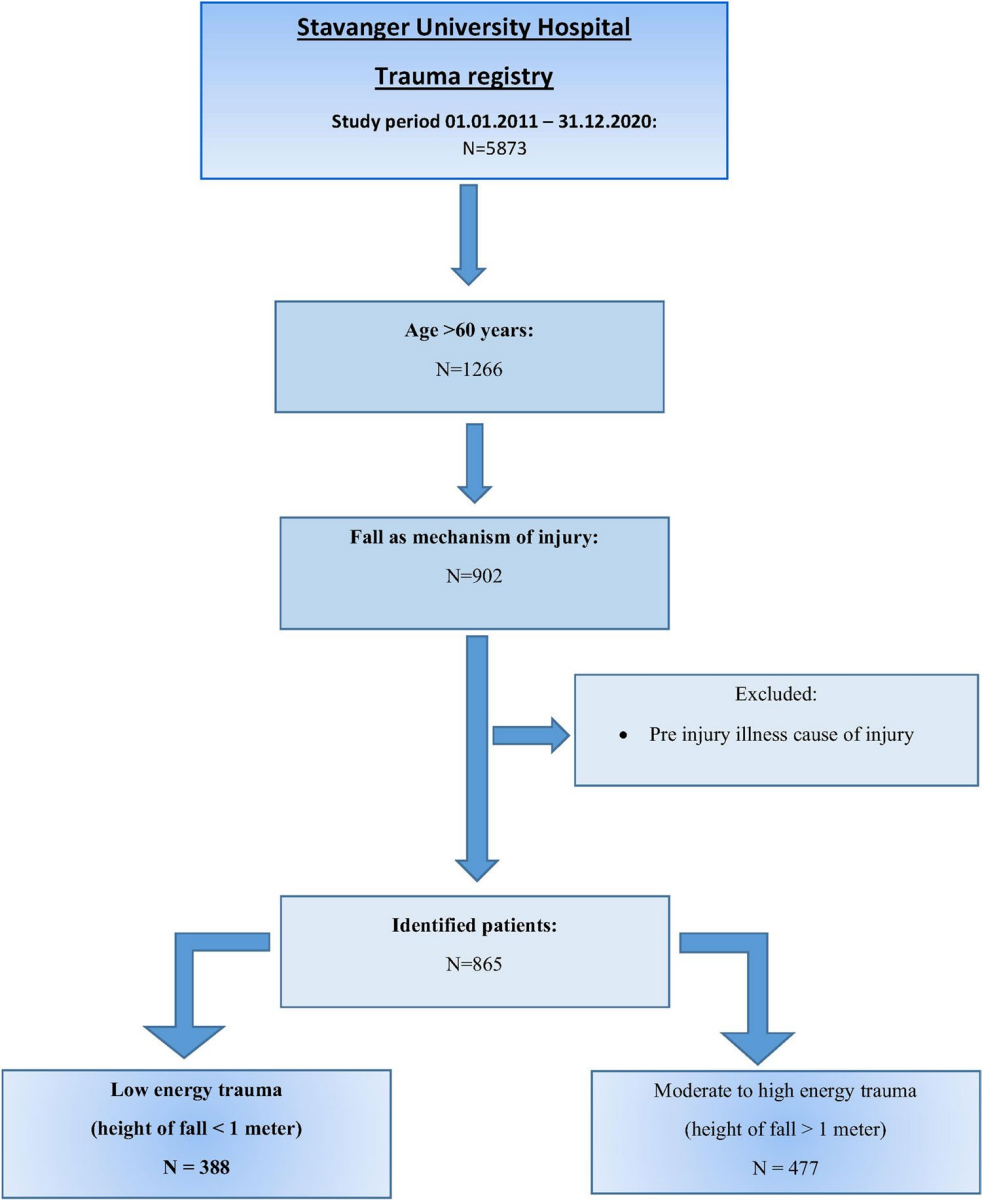
Flow chart of the study group

During the 10-year study period, 5873 patients were registered in the SUH trauma registry. More than 20% (1266 of 5873) were > 60 years, and 71% (902 of 1266) of the patients aged > 60 years were admitted with fall as mechanism of injury. Almost half (388 of 902; 43%) of the patients aged > 60 years with fall injury fell from height less than 1 m (low energy fall) and were included for further analyses (Fig. 1).

The study sample (*n* = 388) consisted of 206 (53%) males and 182 (47%) females. Median age was 78 years (IQR 68–86). Major trauma (ISS > 15) was seen in 23% (90 of 386). Pre-injury ASA score was 2 or 3 in 83% (322 of 388). Twenty-seven percent (87 of 312) had GCS ≤ 13. Further baseline characteristics are shown in Table 1.

**Table 1:** Baseline characteristics for included patients, *n* = 388

Characteristic	*n* (%)
Age in years, median (IQR)	78 (68–86)
Male sex	206 (53.1%)
ISS, median (IQR) ^*n*=386^	9 (5–14)
Major trauma (ISS > 15) ^*n*=386^	90 (23.3%)
NISS, median (IQR) ^*n*=388^	12 (6–17)
TRISS, median (IQR) ^*n*=386^	0.96 (0.94–0.98)
ASA score	
ASA 1	23 (5.9%)
ASA 2	103 (26.6%)
ASA 3	219 (56.4%)
ASA 4	43 (11.1%)
Ethanol in blood sample ^*n*=381^	51 (13.4%)
SBP < 90 ^*n*=290^	13 (4.5%)
Heart rate, median (IQR) ^*n*=293^	80 (70–95)
Respiratory rate, median (IQR) ^*n*=235^	20 (16–24)
Respiratory rate > 25 ^*n*=235^	53 (22.6%)
Respiratory rate < 9 ^*n*=235^	3 (1.3%)
GCS, median (IQR) ^*n*=312^	15 (13–15)
GCS ≤ 13 ^*n*=312^	85 (27.2%)
LOMI	
Head	160 (41.2%)
Thorax	40 (10.3%)
Head + thorax	4 (1.0%)
Head + thorax + other	1 (0.3%)
Head + other (not thorax)	26 (6.7%)
Thorax + other (not head)	9 (2.3%)
*Not head or thorax:*	*148 (38.1%)*
Face	14 (3.6%)
Neck	1 (0.3%)
Abdomen/pelvic contents	5 (1.3%)
Spine	23 (5.9%)
Upper extremities	15 (3.9%)
Lower extremities	73 (18.8%)
Other*	17 (3.1%)

Data given as counts (percentage) unless otherwise specified

^*^Other includes hypothermal injuries and one burn injury

Abbreviations: *IQR*, interquartile range; *ISS*, Injury Severity Score; *NISS*, New Injury Severity Score; *TRISS*, Trauma and Injury Severity Score; *ASA*, American Society of Anesthesiologists; *SBP*, systolic blood pressure; *GCS*, Glasgow Coma Scale; *LOMI*, Location of Major Injury

The majority of the patients had head injuries, with Location of Major Injury (LOMI) being head injury only in 41% of the cases, and additionally, 8% (31 of 384) having head + other injuries. Lower extremity was the only LOMI in 19% (73 of 384), and 10% (40 of 384) had thorax as the only LOMI.

Among the 90 patients with major trauma, undertriage was seen in 43 of them (48%; 95% CI, 38–58%). Overtriage was seen in 204 of the 296 (69%; 95% CI, 63–74%) patients with ISS ≤ 15. Factors related to undertriage among those with major trauma were ISS, NISS, TRISS, and GCS, i.e., those undertriaged had lower ISS/NISS and higher TRISS and GCS than those who got TTA (see Table 2). Overtriaged patients were more often males, had lower ISS/NISS and higher TRISS, and had more frequently ethanol, GCS ≤ 13, and respiratory rate > 25 than those without major trauma that were appropriately triaged (no TTA).

**Table 2:** Subgroups as shown in Box 1, with characteristics and physiological values, *n* = 386

	Group 1 ISS ≤ 15 and no TTA (*n* = 92)	Group 2 ISS ≤ 15 and TTA (*n* = 204)	*p*-value 1 vs 2	Group 3 ISS > 15 and no TTA (*n* = 43)	Group 4 ISS > 15 and TTA (*n* = 47)	*p*-value 3 vs 4
Age in years, median (IQR)	81 (71–88)	78 (67–86)	0.037	77 (72–87)	75 (65–81)	0.097
Male sex	32 (34.8%)	115 (56.4%)	0.001	26 (60.5%)	31 (66.0%)	0.59
ISS, median (IQR)	10 (9–10)	5 (4–9)	< 0.001	17 (17–24)	25 (17–26)	0.014
NISS, median (IQR)	13 (10–14)	6 (4–11)	< 0.001	24 (18–29)	29 (21–43)	0.037
TRISS, median (IQR)	0.96 (0.96–0.97)	0.97 (0.96–0.98)	< 0.001	0.94 (0.88–0.94)	0.84 (0.53–0.92)	< 0.001
ASA score			0.87			0.80
ASA 1	6 (6.5%)	15 (7.4%)		1 (2.3%)	1 (2.1%)	
ASA 2	25 (27.2%)	55 (27.0%)		12 (27.9%)	11 (23.4%)	
ASA 3	52 (56.5%)	115 (56.4%)		23 (53.5%)	28 (59.6%)	
ASA 4	9 (9.8%)	19 (9.3%)		7 (16.3%)	7 (14.9%)	
Ethanol in blood sample	6 (6.6%) ^*n*=91^	32 (16.1%) ^*n*=199^	0.026	6 (14.3%) ^*n*=42^	7 (14.9%)	0.94
SBP < 90	1 (2.0%) ^*n*=50^	9 (5.1%) ^*n*=176^	0.35	0 (0.0%) ^*n*=29^	3 (8.8%) ^*n*=34^	0.10
Heart rate, median (IQR)	82 (70–95) ^*n*=50^	80 (69–94) ^*n*=177^	0.80	82 (80–90) ^*n*=29^	80 (68–100) ^*n*=36^	0.59
Resp rate, median (IQR)	18 (16–22) ^*n*=40^	20 (16–26) ^*n*=146^	0.12	18 (16–22) ^*n*=19^	22 (16–26) ^*n*=29^	0.26
Resp rate > 25	4 (10.0%)	38 (26.0%)	0.032	2 (10.5%)	8 (27.6%)	0.16
Resp rate < 9	0 (0.0%)	2 (1.4%)	0.46	0 (0.0%)	1 (3.5%)	0.41
GCS, median (IQR)	15 (15–15) ^*n*=53^	15 (14–15) ^*n*=187^	0.001	15 (14–15) ^*n*=30^	12 (7–14) ^*n*=40^	< 0.001
GCS ≤ 13	5 (9.4%)	45 (24.1%)	0.021	7 (23.3%)	27 (67.5%)	< 0.001
LOMI head/involving head	31 (33.7%)	92 (45.1%)	0.065	33 (76.7%)	35 (74.5%)	0.80
LOMI thorax/involving thorax	15 (16.3%)	27 (13.2%)	0.48	6 (14.0%)	5 (10.6%)	0.63

Data presented as count (percentage) unless otherwise specified

Abbreviations: *IQR*, interquartile range; *ISS*, Injury Severity Score; *NISS*, New Injury Severity Score; *TRISS*, Trauma and Injury Severity Score; *ASA*, American Society of Anesthesiologists; *SBP*, systolic blood pressure; *GCS*, Glasgow Coma Scale; *LOMI*, Location of Major Injury

Outcome measures such as discharge destination, in hospital mortality, and 30-day mortality were analyzed and sorted in subgroups in Table 3. For the total study group, the 30-day mortality was 13%, while the undertriaged group had a 30-day mortality of 26%. Fifty percent of the total study group was discharged to home, 31% to nursing home, 9% in hospital mortality, and the remaining 10% were transferred to other hospitals or rehabilitation facilities.

**Table 3:** Discharge destination and mortality of the total study group and subgroups

	Total (*n* = 388)	Group 1 ISS ≤ 15 and no TTA (*n* = 92)	Group 2 ISS ≤ 15 and TTA (*n* = 204)	Group 3 ISS > 15 and no TTA (*n* = 43)	Group 4 ISS > 15 and TTA (*n* = 47)
*30 days mortality*	51 (13.2%) ^*n*=386^	6 (6.5%)	14 (6.9%) ^*n*=202^	11 (25.6%)	20 (42.6%)
In hospital mortality	35 (9.0%)	2 (2.2%)	9 (4.4%)	6 (14.0%)	18 (38.3%)
Transfer to other hospital, higher level	5 (1.3%)	-	4 (2.0%)	1 (2.3%)	-
Transfer to other hospital, same level	8 (2.1%)	2 (2.2%)	3 (1.5%)	-	3 (6.4%)
Discharge to home	194 (50.0%)	48 (52.2%)	113 (55.4%)	20 (46.5%)	11 (23.4%)
Transfer to rehabilitation facility	24 (6.2%)	5 (5.4%)	8 (3.9%)	4 (9.3%)	7 (14.9%)
Transfer to nursing home	119 (30.7%)	35 (38.0%)	64 (31.4%)	12 (27.9%)	8 (17.0%)
Missing	3 (0.8%)	-	3 (1.5%)	-	-

Two observations were not possible to classify into groups, i.e., the sum of group sizes is 386

## Discussion

In this study, we found that patients aged > 60 years with low energy falls suffer from head injuries in about half the cases. The undertriage rate was tenfold higher than accepted, at almost 50%, and GCS ≤ 13 was noted in 1 of 4 patients. The 30-day mortality of the total study group was 13%, and 26% in the undertriaged group.

Elderly patients differ from the younger population as they often have a higher degree of multimorbidity and frailty [19, 20]. They have less physiological reserve and may be prone to polypharmacy [21]. Anticoagulative drugs, beta blockers, and steroids can potentially influence the physiologic response to trauma and may mask the severity of injury [21, 22], while other medications such as hypnotics, analgesics, and sedatives make this patient group more prone to falls with subsequent injury [23, 24].

Several studies have found that elderly trauma patients have increased mortality, even with minor or moderate injuries [25, 26]. Major trauma patients benefit from a trauma team activation protocol, and patients that are not met by a trauma team may experience a delay in diagnostics and treatment that could potentially lead to worse outcome. Recent studies have shown that patients with higher age, and those with fall as mechanism of injury, are less likely to be met by a trauma team [10, 27]. The undertriage rate is essential to investigate and should be reduced to a minimum, where an undertriage rate of 5% or less should be the goal.

Age > 60 years is a relative trauma criteria in current national guidelines [28], but does not lead to a trauma team activation in itself; thus, uncertainties may arise for the prehospital teams that initially meet the patient. Recent studies have shown that the criteria of triage recommended from the American College of Surgeons in many cases fail to identify geriatric trauma patients [25]. Adjustments of the triage criteria are proposed to make them more sensitive to changes in the physiology of the elderly (such as systolic blood pressure), to focus on specific centers trained to take care of the elderly, and to optimize the triage and treatment of these patients [29–31]. It has also been suggested that age over 70 years alone should be a criterion for trauma team activation, and that additionally, early intensive monitoring, evaluation, and resuscitation of elderly trauma patients may improve survival [32].

Prevention of falls is another aspect that needs enhanced focus in the aging population. Simple measures like a fall prevention tool kit reduced falls in the elderly with 15% [33]. The Center for Disease Control and Prevention’s (CDC) Stopping Elderly Accidents, Deaths and Injuries (STEADI) initiative [34, 35] pinpoint several modifiable risk factors for the prevention of falls in the elderly. This underlines that prevention is possible and preferred to treatment.

A high degree of overtriage of about 70% was also seen in this study, but is in line with and even lower than the overtriage in the general trauma population at our hospital and most Norwegian centers, where the overtriage usually is about 80%. Overtriage is a problem by allocating resources to the wrong patients and also by increasing fatigue to the trauma team. Hence, reducing overtriage is important and is part of a current project in our hospital.

Resources and economical aspects should be considered as well as ethical points of view regarding when to set the limit of interventions and hospitalizations for the elderly. Still, it is important both to educate surgeons in treatment of the elderly trauma patients and to organize good tenets of trauma resuscitation at the trauma centers [25]. Trauma resuscitation must be dedicated to the physiological and anatomical challenges in the older patient population.

The findings in the previous [10] and current study from our group have led us to implement a geriatric trauma team on demand to possibly improve the care of elderly patients with low energy falls. We plan to do further analyses of before and after implementation of this geriatric trauma team to evaluate potential effects on treatment, outcome, mortality, and under and overtriage.

### Study limitations

Even though the study is based on a prospectively maintained trauma registry, the study is retrospective in nature with its implied limitations. Also, it may be possible that some of the patients with low energy falls could have been admitted through the outpatient clinic of acute injuries without being included in the data of the emergency department, thus not identified by our registrars and missed.

## Conclusion

We include almost 40 patients with age > 60 and low energy falls in our trauma registry yearly. This trauma patient group is dominated by head injuries, and the 30-day mortality rate is 13%. Patients with major trauma (ISS > 15) are undertriaged in half the cases mandating increased awareness of this patient group.

Parts of this study have been presented at the 22nd European Congress of Trauma and Emergency Surgery in May 2023, Ljubljana, Slovenia.

## Data Availability

The data presented in this study can be available on request.

## References

[1] van Laarhoven JJ, Lansink KW, van Heijl M, Lichtveld RA, Leenen LP (2014). Accuracy of the field triage protocol in selecting severely injured patients after high energy trauma. Injury.

[2] Sasser SM, Hunt RC, Faul M, Sugerman D, Pearson WS, Dulski T, et al. Guidelines for field triage of injured patients recommendations of the National Expert Panel on Field Triage, 2011. MMWR Recomm Rep 2012;61(Rr-1):1–20 22237112

[3] Trauma ACoSCo. Resources for optimal care of the injured patient. 2014. pp 24–28.

[4] Haagsma JA, Charalampous P, Ariani F, Gallay A, Moesgaard Iburg K, Nena E (2022). The burden of injury in Central, Eastern, and Western European sub-region: a systematic analysis from the Global Burden of Disease 2019 Study. Arch Public Health.

[5] Støen RO, Nordsletten L, Meyer HE, Frihagen JF, Falch JA, Lofthus CM (2012). Hip fracture incidence is decreasing in the high incidence area of Oslo. Norway Osteoporos Int.

[6] Cuevas-Østrem M, Røise O, Wisborg T, Jeppesen E. Epidemiology of geriatric trauma patients in Norway: a nationwide analysis of Norwegian Trauma Registry data, 2015–2018. A retrospective cohort study. Injury. 2020;52(3):450–9. 10.1016/j.injury.2020.11.007. 10.1016/j.injury.2020.11.007 33243523

[7] Evans D, Pester J, Vera L, Jeanmonod D, Jeanmonod R (2015). Elderly fall patients triaged to the trauma bay: age, injury patterns, and mortality risk. Am J Emerg Med.

[8] Rehn M, Eken T, Krüger AJ, Steen PA, Skaga NO, Lossius HM (2009). Precision of field triage in patients brought to a trauma centre after introducing trauma team activation guidelines. Scand J Trauma Resusc Emerg Med.

[9] Lee H, Bein KJ, Ivers R, Dinh MM (2015). Changing patterns of injury associated with low-energy falls in the elderly: a 10-year analysis at an Australian Major Trauma Centre. ANZ J Surg.

[10] Thorsen K, Narvestad JK, Tjosevik KE, Larsen JW, Soreide K (2022). Changing from a two-tiered to a one-tiered trauma team activation protocol: a before-after observational cohort study investigating the clinical impact of undertriage. Eur J Trauma Emerg Surg..

[11] Lossius HM, Rehn M, Tjosevik KE, Eken T (2012). Calculating trauma triage precision: effects of different definitions of major trauma. J Trauma Manag Outcomes.

[12] Baker SP, O'Neill B, Haddon W, Long WB (1974). The injury severity score: a method for describing patients with multiple injuries and evaluating emergency care. J Trauma.

[13] Association for the Advancement of Automotive Medicine. 2016–2024. https://www.aaam.org/abbreviated-injury-scale-ais/about-ais/.

[14] Cuschieri S (2019). The STROBE guidelines. Saudi J Anaesth.

[15] Palmer CS, Gabbe BJ, Cameron PA (2016). Defining major trauma using the 2008 Abbreviated Injury Scale. Injury.

[16] Roden-Foreman JW, Rapier NR, Yelverton L, Foreman ML (2018). Avoiding Cribari gridlock: the standardized triage assessment tool improves the accuracy of the Cribari matrix method in identifying potential overtriage and undertriage. J Trauma Acute Care Surg.

[17] Samin OA (1999). The new injury severity score versus the injury severity score in predicting patient outcome: a comparative evaluation on trauma service patients of the Auckland Hospital. Ann Adv Automot Med.

[18] Sullivan KM. Open Epi: version 3.01. 2013. https://www.openepi.com/Proportion/Proportion.htm.

[19] Cesari M, Pérez-Zepeda MU, Marzetti E (2017). Frailty and multimorbidity: different ways of thinking about geriatrics. J Am Med Dir Assoc.

[20] Morley JE, Vellas B, van Kan GA, Anker SD, Bauer JM, Bernabei R (2013). Frailty consensus: a call to action. J Am Med Dir Assoc.

[21] Mortazavi SS, Shati M, Keshtkar A, Malakouti SK, Bazargan M, Assari S (2016). Defining polypharmacy in the elderly: a systematic review protocol. BMJ Open.

[22] Dovjak P (2022). Polypharmacy in elderly people. Wien Med Wochenschr.

[23] Pfortmueller CA, Lindner G, Exadaktylos AK (2014). Reducing fall risk in the elderly: risk factors and fall prevention, a systematic review. Minerva Med.

[24] Ambrose AF, Paul G, Hausdorff JM (2013). Risk factors for falls among older adults: a review of the literature. Maturitas.

[25] Adams SD, Holcomb JB (2015). Geriatric trauma. Curr Opin Crit Care.

[26] Bakke HK, Dehli T, Wisborg T (2014). Fatal injury caused by low-energy trauma - a 10-year rural cohort. Acta Anaesthesiol Scand.

[27] Benhamed A, Fraticelli L, Claustre C, Gossiome A, Cesareo E, Heidet M (2023). Risk factors and mortality associated with undertriage after major trauma in a physician-led prehospital system: a retrospective multicentre cohort study. Eur J Trauma Emerg Surg..

[28] traumatologi NKf. Traumeplan NKT. 2020. https://traumeplan.no/index.php?action=showtopic&topic=mxkjMqkD.

[29] Bradburn E, Rogers FB, Krasne M, Rogers A, Horst MA, Beelen MJ (2012). High-risk geriatric protocol: improving mortality in the elderly. J Trauma Acute Care Surg.

[30] Heffernan DS, Thakkar RK, Monaghan SF, Ravindran R, Adams CA, Kozloff MS (2010). Normal presenting vital signs are unreliable in geriatric blunt trauma victims. J Trauma.

[31] Brown JB, Gestring ML, Forsythe RM, Stassen NA, Billiar TR, Peitzman AB (2015). Systolic blood pressure criteria in the National Trauma Triage Protocol for geriatric trauma: 110 is the new 90. J Trauma Acute Care Surg.

[32] Demetriades D, Karaiskakis M, Velmahos G, Alo K, Newton E, Murray J (2002). Effect on outcome of early intensive management of geriatric trauma patients. Br J Surg.

[33] Dykes PC, Burns Z, Adelman J, Benneyan J, Bogaisky M, Carter E (2020). Evaluation of a patient-centered fall-prevention tool kit to reduce falls and injuries: a nonrandomized controlled trial. JAMA Netw Open.

[34] Moreland B, Kakara R, Henry A (2020). Trends in nonfatal falls and fall-related injuries among adults aged ≥65 years - United States, 2012–2018. MMWR Morb Mortal Wkly Rep.

[35] Lee R (2017). The CDC's STEADI initiative: promoting older adult health and independence through fall prevention. Am Fam Physician.

